# Employment Uncertainty and Mental Health During the COVID-19 Pandemic Initial Social Distancing Implementation: a Cross-national Study

**DOI:** 10.1007/s40609-020-00201-4

**Published:** 2021-01-07

**Authors:** Mary Ruffolo, Daicia Price, Mariyana Schoultz, Janni Leung, Tore Bonsaksen, Hilde Thygesen, Amy Østertun Geirdal

**Affiliations:** 1 School of Social Work, University of Michigan, 1080 South University, Ann Arbor, MI 48109 USA; 2 School of Social Work, University of Michigan, 1080 South University, Ann Arbor, MI 48109 USA; 3 Faculty of Health and Life Sciences, Northumbia University, Manor House, Coach LanNE7 & 7TR, Newcastle On the Tyne, UK; 4 Faculty of Health and Behavioural Science, The University of Queensland, 17 Upland Roasm, St. Lucia, QLD 4067 Australia; 5 Faculty of Social and Health Sciences, Department of Health and Nursing Sciences, Inland Norway University of Applied Sciences, Elverum, Norway; 6 Faculty of Health Studies, VID Specialized University, Sandnes, Norway; 7 Faculty of Health Sciences, Department of Occupational Therapy, Prosthetics and Orthotics, Oslo Metropolitan University, PO Box 4 St Olavs Plass, 0130 Oslo, Norway; 8 Faculty of Social Sciences, Department of Social Work, Oslo Metropolitan University, Oslo, Norway

**Keywords:** COVID-19, Coronavirus, Economic uncertainty, Employment, Mental health, Social distancing

## Abstract

**Background:**

Social distancing during the COVID-19 pandemic has resulted in changes in the work environment and employment uncertainty. This paper reports on a cross-national comparison of four countries (Norway, UK, USA and Australia) and examines the differences in mental health between those individuals employed and those not employed during the social distancing implementation.

**Methods:**

Participants (*N* = 3,810) were recruited through social media in April/May 2020 and were invited to complete a self-administered electronic survey over a 3-week period. Differences between those employed and those not employed with regard to their sociodemographic characteristics and mental health were investigated with chi-square tests, independent *t* tests, and one-way analysis of variances (ANOVAs).

**Results:**

Compared with their counterparts, participants who were employed reported lower levels of mental health distress (*p* < 0.001), higher levels of psychosocial well-being (*p* < 0.001), better overall quality of life (*p* < 0.001), and lower levels of overall loneliness, social loneliness, and emotional loneliness (*p* < 0.001). Small to medium but consistent differences (Cohen’s *d* = 0.23–0.67) in mental health favor those with employment or those who were retired.

**Conclusion:**

Further study is needed to assess mental health over time as the COVID-19 pandemic and employment uncertainty continues.

## Introduction

During the initial social distancing protocol implementation for the novel coronavirus (COVID-19) pandemic in February/March 2020, many people experienced changes in the work environment (e.g., increase in remote work options), job uncertainty, and job loss (Douglas et al., 2020). The move to use social distancing as a primary way to slow the spread of the COVID-19 pandemic and reduce the risk that health care systems would be overwhelmed was a major policy decision adopted by many countries across the world (WHO, 2020). The obvious benefit of the policy decision was to mitigate the effects of COVID-19 spread, but this response can impact the mental health of individuals experiencing social isolation, as well as job changes, job loss, and job uncertainty.

Social distancing protocols included individuals self-isolating at home if experiencing COVID-19 symptoms; closure of “non-essential” workplaces; bans on large gatherings; closure of daycares, schools, and universities; and limiting contact with special populations who are at higher risk of poor health outcomes if infected with COVID-19 (WHO, 2020).

Employment uncertainty and economic changes create high risk for the overall mental well-being of individuals and families (Holland, 2016; Jonsson et al., 2020), and lessons from past crisis situations suggest that not all members of society are equally affected by the crisis (Godinc et al., 2019). Social isolation, economic uncertainty, and limited access to health care and adequate essential services are factors that may affect how severely the psychological and mental health of individuals are impacted during times of crisis (Godinc et al., 2019). The COVID-19 pandemic, with the adoption of social distancing guidelines, resulting in “lockdowns” and “shelter in place” orders for many communities and “quarantines” for infected individuals, changed suddenly the way people organized work and social relationships. These changes in work schedules and social interactions can affect the coping mechanisms and the general sense of well-being of individuals. High rates of anxiety, depression, post-traumatic stress disorder, psychological distress, and stress were reported in the general population during the COVID-19 pandemic in China, Spain, Italy, Iran, United States of America (USA), Turkey, Nepal, and Denmark (Xiong et al., 2020). In a study completed in the USA, greater job insecurity due to the COVID-19 pandemic was related to greater depressive symptoms and indirectly related to greater anxiety symptoms due to increased financial concerns (Wilson et al., 2020).

Study aim: The aim of this study was to examine the differences in mental health between those individuals employed and those not employed during the initial phases of the social distancing implementation during the COVID-19 pandemic.

## Methods

Setting: The study is a cross-national comparison of four countries (Norway, United Kingdom (UK), United States of America (USA), and Australia). Utilizing electronic communication, via different social media such as Facebook, and Instagram, individuals from the general population were invited in April/May 2020 to complete a self-administered survey over a 3-week period. Each country had a landing site for the survey at the involved universities. These were Oslo Metropolitan University, Norway; University of Michigan, USA; University of Salford, UK; and University of Queensland, Australia. The survey was translated from Norwegian to English by the researchers according to language and cultural contexts.

Participants: Individuals that were 18 years of age and over were invited to complete the self-administered survey online that was monitored by corresponding academic institutions. The participants needed to understand Norwegian or English and be living in one of the four countries at the time of the survey.

Sociodemographic variables included employment status (full-time/part-time or not employed), living area, age group, gender, highest completed education, cohabitation, living with children, and type of work setting.

Measures: The measures included four questionnaires/scales that capture the experiences of participants related to mental health. General Health Questionnaire (GHQ-12) is a self-report measure of mental health (Goldberg et al., 1997; Goodwin et al., 2013). The support for the validity of this measure has been demonstrated in several large studies of the general adult population, clinical populations, and work populations (Aalto et al., 2012; Adlaf et al., 2001; Firth, 1986; Goodwin et al., 2013; Gorter et al., 2008). Six items of the GHQ-12 are phrased as a positive experience (e.g., “able to enjoy day to day activities), while six items are phrased as a negative experience (e.g., “felt constantly under strain”). On each item, the person indicates the degree to which the item content has been experienced during the two preceding weeks, using four response categories (“less than usual,” “as usual,” “more than usual,” or “much more than usual”). Items are scored between 0 and 3, and positively formulated items are recoded prior to analysis. As a result, the GHQ-12 scale score range is 0–36, with higher scores indicating poorer mental health (more psychological distress (e.g., high levels of anxiety, depression, stress). Psychosocial well-being (PSW) assesses an individual’s psychological experience of well-being and consists of ten items. The measure includes five positive and five negative statements, and scores on the total scale are between 1 (highest well-being) and 5 (lowest well-being) (Kaasa et al., 1988). Positively worded items are reversed and then the total score is calculated by averaging the scores on the 10 items. The Six-Item De Jong Gierveld Loneliness Scale (Gierveld & Tilburg, 2006) consists of six statements, all of which are rated from 0 (totally disagree) to 4 (totally agree). This scale is designed to measure two different aspects of loneliness, emotional loneliness, and social loneliness. Emotional loneliness addresses feelings of emptiness and rejection, while social loneliness focuses on having people around and people to rely on. A previous factor-analysis study found that the six statements loaded on two different factors, and therefore they should be treated as constituting two different scales reflecting the two different aspects of loneliness (Gierveld & Tilburg, 2006). A higher sum score of all scale items would indicate higher overall loneliness. Cantril’s Ladder (CL) is a self-administered overall quality-of-life questionnaire with one question, “How is your life?” asking the respondent to rate his or her present experience of life on a scale anchored by their own identified values. The response alternatives are between 0 and 10 with 0 corresponding to the worst possible quality of life and 10 corresponding to the best possible quality of life (Cantril, 1965). A cut-off score of 6 was set to represent a minimally good overall quality of life (Cantril, 1965). The CL has been reported to have good validity and stability and reasonable reliability (Atkinsen, 1982; Jenkins et al., 2005).

Statistical analysis: The overall sample and each country subsamples of those employed and those not employed were analyzed with frequencies and percentages for categorical variables. The chi-square test was used to analyze overall differences in proportions between groups. For continuous variables, means and standard deviations were used to describe the variables. Differences in mental health (mental health distress (GHQ-12), psychosocial well-being (PSW), loneliness (emotional loneliness, social loneliness, overall loneliness), and quality of life (CL)) between participants who were employed and those who were not employed were analyzed using independent *t* tests. Effect sizes were calculated using Cohen’s *d*. According to Cohen (1992), a small effect size is about 0.20, a medium effect size is about 0.50, and a large effect size is about 0.80 or above. The effect size calculation captures the standardized mean difference between two groups. One-way analyses of variance (ANOVAs) were conducted for participants who were employed by country and by educational level. The dependent measures included the four measures of mental health. Post hoc analyses used Bonferroni post hoc test.

Ethics: The data collected in this self-administered survey was anonymous. Each of the four universities’ ethics or institutional review boards approved the study. For Norway, the review was conducted by Oslo Metropolitan University and the Regional Committee for Medical and Health Research Ethics (REK; project reference 132,066). In the USA, it was reviewed by the University of Michigan Institutional Review Board for Health Sciences and Behavioral Sciences (IRB HSBS) and designated as exempt (UHUM00180296). In the UK, the study was reviewed by the University Research Ethics (HSR1920-080) and in Australia by the University of Queensland Human Research Ethics Office (HSR 2020000956).

## Results

Participants: The sociodemographic characteristics of the total sample by employed and not employed status and by country are displayed in Table 1. There were a total of 1117 (29.3%) participants who reported being not employed and 2692 (70.7%) participants who reported being employed full time or part time. Within each country, differences emerged between participants employed and those who were not employed in age and educational levels, consistent with the total sample results. Participants between the ages of 30 to 59 were more likely to be employed when compared with participants younger than 30 or over 59 years. Participants with a bachelor’s degree or higher were more likely to be employed in full-time or part-time jobs.

**Table 1:** Sociodemographic characteristics for total sample and by country

		Total sample	*p*	Norway	*p*	UK	*p*	USA	*p*	Australia	*p*
Status			* < 0.001*								
	Not employed	1,117 (29.3%)		125 (16.2%)		408 (29.7%)		513 (36.9%)		71 (26%)	
	Employed	2,692 (70.7%)		646 (83.8%)		965 (70.3%)		879 (63.1%)		202 (74%)	
Living area			* < 0.001*		*NS*		* < 0.002*		* < 0.008*		*NS*
Rural/Farming	Not employed	96 (34%)		9 (14.3%)		24 (31.6%)		61 (44.2%)		2 (40%)	
	Employed	186 (66%)		54 (85.7%		52 (68.4%)		77 (55.8%)		3 (60%)	
Small Town	Not employed	277 (32.9%)		24 (16%)		69 (29.6%)		175 (41.6%)		9 (23.1%)	
	Employed	566 (67.1%)		126 (84%)		164 (70.4%)		246 (58.4%)		30 (76.9%)	
Medium-size city	Not employed	393 (32%)		15 (12.9%)		176 (35.3%)		189 (33.2%)		13 (29.5%)	
	Employed	835 (68%)		101 (87.1%)		322 (64.7%)		381 (66.8%)		31 (70.5%)	
Large city	Not employed	351 (24.1%)		77 (17.4%)		139 (24.6%)		88 (33.5%)		47 (25.4%)	
	Employed	1,105 (75.9%)		365 (82.6%)		427 (75.4%)		175 (66.5%)		138 (74.6%)	
Age group			* < 0.001*		* < 0.001*		* < 0.001*		* < 0.001*		* < 0.001*
18–29	Not employed	214 (30.4%)		45 (23.9%)		71 (35.3%)		78 (32.4%)		20 (26.7%)	
	Employed	491 (69.6%)		143 (76.1%)		130 (64.7%)		163 (67.6%)		55 (73.3%)	
30–39	Not employed	127 (17.8%)		19 (10.8%)		56 (23.7%)		45 (18.4%)		7 (12.5%)	
	Employed	586 (82.2%)		157 (89.2%)		180 (76.3%)		200 (81.6%)		49 (87.5%)	
40–49	Not employed	116 (14%)		16 (8.1%)		43 (12.4%)		53 (22.1%)		4 (9.5%)	
	Employed	710 (86%)		182 (91.9%)		303 (87.6%)		187 (77.9%)		38 (90.5%)	
50–59	Not employed	149 (20.6%)		7 (6%)		66 (20.8%)		66 (27.2%)		10 (21.3%)	
	Employed	574 (79.4%)		109 (94%)		251 (79.2%)		177 (72.8%)		37 (78.7%)	
60–69	Not employed	320 (52.3%)		19 (26.8%)		116 (55.5%)		163 (56.2%)		22 (52.4%)	
	Employed	292 (47.4%)		52 (73.2%)		93 (44.5%)		127 (43.8%)		20 (47.6%)	
70 plus	Not employed	187 (83.5%)		19 (86.4%)		56 (87.5%)		104 (81.9%)		8 (72.7%)	
	Employed	17 (16.5%)		3 (13.6%)		8 (12.5%)		23 (18.1%)		3 (27.3%)	
Gender			*NS*		*NS*		*NS*		*NS*		*NS*
Male	Not employed	227 (31.6%)		29 (20.3%)		57 (28.8%)		128 (39.5%)		13 (24.5%)	
	Employed	491 (68.4%)		114 (79.7%)		141 (71.2%)		196 (60.5%)		40 (75.5%)	
Female	Not employed	870 (28.7%)		96 (15.3%)		347 (29.9%)		371 (35.8%)		56 (26.5%)	
	Employed	2163 (71.3%)		532 (84.7%)		812 (70.1%)		664 (64.2%)		155 (73.5%)	
Education level			* < 0.001*		* < 0.001*		* < 0.001*		* < 0.001*		* < 0.003*
Elementary	Not employed	12 (70.6%)		6 (66.7%)		0 (0%)		2 (66.7%)		4 (80%)	
	Employed	5 (29.4%)		3 (33.3%)		0 (0%)		1 (33.3%)		1 (20%)	
High School	Not employed	158 (41.6%)		36 (32.1%)		44 (34.6%)		73 (61.3%)		5 (22.7%)	
	Employed	222 (58.4%)		76 (67.9%)		83 (65.4%)		46 (38.7%)		17 (77.3%)	
Technical/Associate Degree	Not employed	243 (41%)		11 (42.3%)		122 (36.9%)		89 (50.9%)		21 (35%)	
	Employed	349 (59%)		15 (59.7%)		209 (63.1%)		86 (49.1%)		39 (65%)	
Bachelor’s Degree	Not employed	361 (28.6%)		39 (14.3%)		137 (29.7%)		166 (35.4%)		19 (32.2%)	
	Employed	901 (71.4%)		233 (85.7%)		325 (70.3%)		303 (64.6%)		40 (67.8%)	
Master’s/Doctoral Degree	Not employed	343 (22%)		33 (9.4%)		105 (23.2%)		183 ( 29.2%)		22 (17.5%)	
	Employed	1,214 (78%)		319 (90.6%)		348 (76.8%)		442 (70.8%)		104 (82.5%)	
Cohabitation			* < 0.004*		*NS*		* < 0.004*		*NS*		*NS*
No	Not employed	244 (33.7%)		29 (18%)		91 (37.4%)		113 (41.1%)		11 (24.4%)	
	Employed	480 (66.3%)		132 (82%)		152 (62.6%)		162 (58.9%)		34 (75.6%)	
Yes	Not employed	872 (26.3%)		96 (15.7%)		317 (28.1%)		399 (35.8%)		60 (26.4%)	
	Employed	2,206 (71.7%)		514 (84.3%)		810 (71.9%)		715 (64.2%)		167 (73.6%)	

When examining the differences related to work changes for those participants who were employed across countries, there emerged a significant difference (*p* < 0.001) between countries on the proportion of participants reported working remotely, being temporarily furloughed or laid off, being fired, continuing to work at the workplace or no change due to COVID-19. For example, only 41.2% of participants who were employed in the UK reported working remotely while over 50% of participants in the USA, Australia, and Norway reported working remotely. Participants differed in the type of work they did, with a higher percentage of participants in Norway (68.8%) and the UK (48.9%) reporting working in the healthcare system when compared with USA (24%) and Australia (29.7%). Table 2 reports these findings.

**Table 2:** Cross country comparison for work situation changes, types of settings and reasons for unemployment

	Norway	UK	USA	Australia	
	*n*/Percent within Country	*n*/Percent within Country	*n*/Percent within Country	*n* /Percent within Country	*p*
Work situation since COVID-19 Pandemic					< 0.001
Working remotely	396/61%	454/41.2%	577/50.3%	117/50.4%	
Temporarily furloughed/laid off -FT job	43/6.6%	115/10.4%	66/5.8%	1/4%	
Temporarily furloughed/laid off-PT job	20/3.5%	111/10.1%	76/6.6%	22/9.5%	
Fired or let go from job	1/.1%	10/.9%	35/3.1%	6/2.6%	
Continue to work at workplace	103/15.8%	204/18.5%	119/10.4%	36/15.5%	
No change	85/13%	208/18.9%	274/23.9%	50/21.6%	
Type of work setting					< 0.001
Healthcare	106/68.8%	259/46.9%	111/24%	39/29.7%	
Trade industry	6/3.9%	10/1.8%	14/3%	5/3.9%	
Construction industry	1/.6%	17/3.1%	12/2.6%	4/3.1%	
Essential functions/jobs	15/9.7%	83/15%	86/18.6%	22/17.2%	
Other job type	26/16.9%	183/33.2%	239/51.7%	59/46.1%	
Reason for not working					< 0.001
Retired	31/25.2	180/41.3%	277/47.9%	24/32%	
Receiving disability benefits	11/8.9%	50/11.5%	32/5.5%	7/9.3%	
On sick leave	8/6.5%	13/3%	9/1.6%	1/1.3%	
Other benefits	6/4.9%	19/4.4%	15/2.6%	6/8%	
Parental leave	12/9.8%	9/2.1%	14/2.4%	3/4%	
Other reasons	55/44.7%	165/37.8%	221/40%	34/45.3%	

There was a significant difference (*p* < 0.001) across countries related to the reason for the unemployment for those participants who were not employed. In terms of participants who reported that they were not employed, almost half of the participants in the UK (41.3%) and the USA (47.9%) were retired, while in Norway (25.2%) and Australia (32%), smaller proportions were retired. To further explore these differences, *t* tests for independent samples between participants who were retired and those participants who were not employed and not retired were performed. Significant differences in means were noted on all the measures of mental health at the *p* < 0.001 level. Medium effect sizes were noted on psychological distress, emotional loneliness, overall loneliness, and quality of life. The results are located in Table 3.

**Table 3:** Mental health between those participants who were retired and those participants who were not employed but not retired

Total Sample	Retired	Not retired and not employed			
	(Mean/*SD*)	(Mean/*SD*)	Mean Difference	Effect size Cohen’s *d*	*p*
Mental health distress	14.74/6.12	18.86	− 4.12	0.60	< 0.001
	*n* = 483	*n* = 536			
Psychosocial well being	2.70/0.41	2.88/0.44	− 0.18	0.42	< 0.001
	*n* = 483	*n* = 536			
Social loneliness	3.79/2.70	4.96/3.22	− 1.75	0.39	< 0.001
	*n* = 476	*n* = 535			
Emotional loneliness	5.27/2.59	7.05/2.65	− 1.78	0.67	< 0.001
	*n* = 477	*n* = 534			
Overall loneliness	9.06/4.13	12.01/4.93	− 2.95	0.64	< 0.001
	*n* = 473	*n* = 534			
Quality of life	6.89/2.18	5.54/2.39	1.34	0.59	< 0.001
	*n* = 483	*n* = 536			

On all measures of mental health, participants who were employed had better mental health than those who were not employed (*p* < 0.001). For two of the measures, social loneliness and overall loneliness, there was a small effect size that emerged (0.249 and 0.232) in the total sample, with those who were employed having lower levels of social and overall loneliness than those not employed. When examining these differences between participants who were employed and those who were not employed by country, there was a small effect size recorded for all the measures of mental health in the Norway subsample between participants who were employed and those who were not employed. In the UK, for the measures of social loneliness, emotional loneliness, overall loneliness, and quality of life, a small effect size was noted (0.21, 0.26, 0.28, 0.36) between participants who were employed and those who were not employed. In the USA subsample, only a few of the mental health measures were statistically significant, but the effect sizes were weak. Similar to Norway and the UK, in Australia, statistically significant differences on most measures with small effect sizes were noted. These findings are located in Table 4.

**Table 4:** Mental health measures by employed and not employed participants by country

Country		Mental health distress	Psychological wellbeing	Social loneliness	Emotional loneliness	Overall loneliness	Quality of life
Norway							
Employed (*n* = 646)	Mean/*SD*	13.17/6.22	2.32/0.26	2.42/2.37	5.07/2.68	7.49/4.32	6.64/1.90
Not employed (*n* = 125)	Mean/*SD*	14.91/7.07	2.58/0.81	3.48/2.8	6.05/2.6	9.51/4.66	6.22/2.09
	Mean difference	1.75	0.26	1.04	0.98	2.02	− 0.61
	Effect size (Cohen’s *d*)	0.26	0.34	0.41	0.37	0.45	0.21
	*P value*	< 0.005	< 0.001	< 0.001	< 0.001	< 0.001	< 0.001
UK							
Employed (*n* = 965)	Mean/*SD*	17.60/7.46	2.83/3.42	4.19/3.04	6.44/ 2.65	10.64/4.69	6.34/2.23
Not Employed (*n* = 408)	Mean/*SD*	19.90/7.64	2.84	4.83/3.11	7.14/ 2.71	11.97/4.79	5.48/2.52
	Mean difference	2.29	0.01	0.63	0.7	1.33	− 0.87
	Effect size (Cohen’s *d*)	0.3	0.03	0.21	0.26	0.28	0.36
	*P value*	< 0.001	0.001	0.001	0.001	0.001	0.001
USA							
Employed (*n* = 879)	Mean/*SD*	16.41/6.01	2.87/0.357	4.17/3.0	6.11/2.44	10.30/4.59	6.48/2.11
Not employed (*n* = 513)	Mean/*SD*	15.33/6.18	2.83/0.339	4.35/3.04	5.67/2.69	10.03/4.68	6.63/2.26
	Mean difference	− 1.08	10.046	0.188	− 0.443	− 0.23	0.183
	Effect size (Cohen’s d)	0.177	0.114	0.059	0.171	0.058	0.058
	*P value*	< 0.001	< 0.01	NS	< 0.05	NS	NS
Australia							
Employed (*n* = 202)	Mean/*SD*	15.74/6.80	2.87/0.313	3.44/2.83	5.44/2.59	10.64/ 4.69	6.34/2.23
Not employed (*n* = 71)	Mean/*SD*	16.89/6.67	2.80/0.335	4.75/3.16	6.20/2.65	11.97/ 4.79	5.48/2.52
	Mean difference	1.15	0.067	1.31	0.762	2.07	0.88
	Effect size (Cohen’s *d*)	0.172	0.216	0.437	0.29	0.416	0.396
	*P value*	NS	NS	< 0.001	< 0.05	< 0.001	< 0.01

One-way analyses of variance showed that participants who were employed significantly differed by country on the self-report of psychological distress (*F*(3, 2687) = 58.58; *p* < 0.001), quality of life (*F*(3, 2688) = 11.02; *p* < 0.001), psychosocial well-being (*F*(3, 2688) = 224.62; *p* < 0.001), and in the measures of overall loneliness (*F*(3, 2688) = 71.20; *p* < 0.001), emotional loneliness (*F*(3, 2677) = 40.47; *p* < 0.001), and social loneliness (*F*(3, 2675) = 60.47; *p* < 0.001). Post hoc analyses showed that employed participants in Norway (*M* = 13.17, *SD* = 6.21) had significantly lower levels of psychological distress than did the employed participants in the UK (*M* = 17.6, *SD* = 7.46, *d* = 0.65), the USA (*M* = 16.41, *SD* = 6.01, *d* = 0.53), and in Australia (*M* = 15.74, *SD* = 6.8, *d* = 0.39). For the quality-of-life measure, employed participants in Norway (*M* = 6.84, *SD* = 1.90) and Australia (*M* = 7.03, *SD* = 2.08) reported a significantly higher mean score on quality of life than did employed participants in the UK (*M* = 6.34, *SD* = 2.29) and the USA (*M* = 6.48, *SD* = 2.11). The employed participants in Norway (*M* = 2.32, *SD* = 0.72) had significantly lower mean scores than the employed participants in UK (*M* = 2.83, *SD* = 0.34), USA (*M* = 2.87, *SD* = 0.31), and Australia (*M* = 2.87, *SD* = 0.51) on measures of psychosocial well-being. In measures of overall loneliness, the employed participants in Norway (*M* = 7.49, *SD* = 4.31) again reported significantly lower levels of overall loneliness when compared with the employed participants in the UK (*M* = 10.64, *SD* = 4.68), USA (*M* = 10.30, *SD* = 4.59), and Australia (*M* = 8.88, *SD* = 4.57). On examination of the emotional loneliness measure, the Norway employed participants (*M* = 5.07, *SD* = 2.68) and the Australian employed participants (*M* = 5.44, *SD* = 2.59) reported significantly lower mean scores than the UK employed participants (*M* = 6.44, *SD* = 2.65) and the US participants (*M* = 6.11, *SD* = 2.44). The US employed participants had significantly lower mean scores on emotional loneliness than did employed participants in the UK. The Norway employed participants (*M* = 2.42, *SD* = 2.36) had significantly lower measures of social loneliness than the employed participants in the UK (*M* = 4.19, *SD* = 3.04), USA (*M* = 4.17, *SD* = 3.00), and Australia (*M* = 3.44, *SD* = 2.83).

The one-way analyses of variance for participants that were employed showed that a higher educational level was significantly associated with lower self-report of psychological distress (*F*(4, 2,685) = 8.20; *p* < 0.001); higher quality of life (*F*(4, 2,686) = 6.57; *p* < 0.001); improved psychosocial well-being (*F*(4, 2,686) = 5.94; *p* < 0.001); and lower levels of loneliness in the measures of overall loneliness (*F*(4, 2,666) = 14.78; *p* < 0.001), emotional loneliness (*F*(4, 2,675) = 15.57; *p* < 0.001), and social loneliness (F(4, 2,673) = 6.85; *p* < 0.001). For the measure of psychological distress, the post hoc analyses showed that employed participants who had an educational level of at least a master’s or doctoral degree (*M* = 15.34, *SD* = 6.41) and those that had a bachelor’s degree (*M* = 16.15, *SD* = 6.93) were significantly different than those with a technical/associate degree (*M* = 17.50, *SD* = 7.34). The higher mean scores for the participants that had a technical/associate degree indicate greater levels of psychological distress when compared with the employed participants with a master’s/doctoral degree or a bachelor’s degree. On the quality-of-life measure, the employed participants with a master’s/doctoral degree (*M* = 6.78, *SD* = 2.0) were on average significantly different from those participants with a technical/associate degree (*M* = 6.25, *SD* = 2.29) or a bachelor’s degree (*M* = 6.42, *SD* = 2.09). This means that the employed participants with a master’s/doctoral degree reported better quality of life when compared with the other two groups. For the psychosocial well-being measure, the employed participants with a master’s/doctoral degree (*M* = 2.69, *SD* = 0.51) were significantly different from those participants with a technical/associate degree (*M* = 2.83, *SD* = 0.41), indicating that those with higher education reported better psychosocial well-being. For overall loneliness, the participants with a high school degree (*M* = 10.53, *SD* = 4.96), technical/associate’s degree (*M* = 10.81, *SD* = 4.89), or bachelor’s degree (*M* = 9.91, *SD* = 4.72) were significantly more likely to report higher levels of overall loneliness than those employed participants with a master’s/doctoral degree (*M* = 8.95, *SD* = 4.54). This same result is true for the emotional loneliness measure with employed participants with a high school degree (*M* = 6.45, *SD* = 2.9), technical/associate’s degree (*M* = 6.53, *SD* = 2.73), or bachelor’s degree (*M* = 6.12, *SD* = 2.62) having significantly higher reports of emotional loneliness when compared with employed participants with a master’s/doctoral degree (*M* = 5.52, *SD* = 2.52). For the social loneliness measure, the employed participants with a master’s/doctoral degree (*M* = 3.42, *SD* = 2.85) were significantly more likely to report lower levels of social loneliness than were those with a high school degree (*M* = 4.05, *SD* = 3.16) or a technical/associate’s degree (*M* = 4.26, *SD* = 3.17).

Using one-way analyses of variance, there emerged significant differences among participants who were employed, based on their experiences working remotely, being furloughed or fired, continuing to work or working with very little changes in their work situation, with respect to the psychological distress measure (*F*(3, 2683) = 23.45, *p* < 0.001), the psychosocial well-being measure (*F*(3, 2684) = 7.47, *p* < 0.001), the loneliness measures (overall loneliness (*F*(3, 2664) = 12.58, *p* < 0.001), social loneliness (*F*(3, 2671) = 5.25, *p* < 0.001), emotional loneliness (*F*(3, 2673) = 17.59, *p* < 0.001), and the quality-of-life measure (*F*(3, 2684) = 11.21, *p* < 0.001). In the post hoc analyses, participants who were employed and worked remotely during the COVID-19 period reported significantly lower levels of psychological distress (*M* = 15.87, *SD* = 6.59) than did participants who were furloughed/laid off (*M* = 18.26, *SD* = 7.59), fired (*M* = 23.33, *SD* = 7.64), or continued to work in their settings with few changes (*M* = 15.08, *SD* = 6.78) at the 0.05 level. On the psychosocial well-being measure, participants who worked remotely (*M* = 2.71, *SD* = 0.53) or who continued to work with little changes to their work setting (*M* = 2,82, *SD* = 0.48) had significantly lower mean difference scores than did those who were furloughed/laid off (*M* = 2.82, *SD* = 0.48) or fired (*M* = 3.08, SD = 0.34) at the 0.05 level. For the loneliness measure, the participants who were furloughed/laid off from their job reported on average significantly higher levels of overall loneliness (*M* = 10.89, *SD* = 4.95), social loneliness (*M* = 4.18, *SD* = 3.09), and emotional loneliness (*M* = 6.71, *SD* = 2.71) than those who reported working remotely (overall loneliness (*M* = 9.43, *SD* = 4.57), social loneliness (*M* = 3.55, *SD* = 2.88), emotional loneliness (*M* = 5.87, *SD* = 2.58)) or had little change in their work setting (overall loneliness (*M* = 9.38, *SD* = 4.83), *SD* = 4.57), social loneliness (*M* = 3.76, *SD* = 3.01), emotional loneliness (*M* = 5.62, *SD* = 2.69)) at the 0.05 level. For the quality-of-life measure, once again if participants could work remotely (*M* = 6.61, *SD* = 2.05) or continue to work in their setting with little change (*M* = 6.72, *SD* = 2.08), they reported higher quality-of-life scores at the 0.05 level than were participants who were furloughed/laid off (*M* = 6.06, *SD* = 2.35) or fired (*M* = 4.92, *SD* = 2.54).

## Discussion

Most participants in this study were employed, lived in medium- to large-size cities, and had a bachelor’s degree or higher levels of education. Across all four countries, participants who were employed demonstrated better mental health than those who were not employed. Being employed was associated with lower levels of overall loneliness, emotional loneliness, social loneliness, and psychological distress and higher levels of psychosocial well-being and quality of life. It appears that individuals who were employed (even if working remotely) remained connected to their employer and colleagues, while individuals who were not employed experienced less interaction and more social isolation. Therefore, in a time of social distancing, people without employment appear to be at risk of experiencing greater levels of psychological distress, loneliness, and low quality of life. This finding is consistent with the literature that suggests having a job provides a certain level of security and functions as a buffer against financial uncertainty and fear (Godinic et al., 2019).

The majority of participants that were employed were working in remote conditions based on social distance protocols being implemented in all four countries. While the employed participants who could work remotely had better measures of mental health than those who were not employed, it will be important to explore how over time this group handles the stresses of working remotely and the loss of engaging with others at the worksite.

While participants who were not employed had poorer mental health, it is too early to assess if the economic aid implemented in each country to address employment loss during the COVID-19 pandemic will change the outcomes on mental health.

It is important to note that for participants working remotely, having this remote option was associated with better mental health outcomes than those participants who experienced little change in their work life. This finding suggests that participants working remotely were able to adjust to this change in positive ways that promoted better mental health outcomes. These participants may not have felt as much employment uncertainty since the type of work they did could be completed away from the work site. When social distancing guidelines are lifted, it will be critical to explore how many participants will seek to continue to work at least some days each week remotely. When examining the mental health outcomes by country, it appears that participants from Norway reported better mental health outcomes than in UK, USA, and Australia. The better mental health outcomes in Norway participants may be related to the fact that participants were more likely to work remotely during the pandemic.

In this study, people who were retired had better overall mental health than those participants that were not employed due to being furloughed, fired, or other reasons. It is possible that participants who are not employed and under 30 years of age may include a mix of younger people who were still studying and not yet in the workforce, or young people who have graduated but were unable to find secure employment. Young people who are seeking employment may feel more discouraged and isolated during the pandemic, which may have resulted in the observed poorer mental health outcomes for this group. Previous studies of young people in the past 5 to 10 years have reported worsening mental health overall (Hunt & Eisengerg, 2010; Mojtabai et al., 2016). Unfortunately, we do not have data on the reasons for this finding but believe that future research is warranted to provide an understanding of how we can better support this vulnerable group. Apart from age groups, future research is warranted to investigate other sociodemographic factors and sub-populations who may be at increased disadvantage from the pandemic.

## Implications for Policy and Practice

This study used a cross-sectional online survey. As with any cross-sectional survey, assumptions about causation cannot be made. This study explored how employment and not working during the early phases of the COVID-19 pandemic and the implementation of social distancing practices were associated with mental health. Many other factors (e.g., disease spread, access to health care services and child care, income, differences in the way social distancing practices were implemented) could also be associated with mental health during this pandemic. Thus, future studies with similar aims and designs may be improved if incorporating these and similar variables as possible confounders. Longitudinal designs may also be incorporated in future studies, which would enable examining whether differences between the groups increase or decrease across time. Gaining increased understanding of how social distancing practices impact mental health and well-being can support development of practice interventions. The sample was recruited through advertisements in social media (e.g., Facebook, Twitter), and the findings may not be generalizable to populations that do not use social media. In addition, many of the participants, under the age of 30 years, may have been students or unemployed prior to COVID-19.

An understanding of how mental health interconnects with employment and various modes of working can support efforts in considering policy, procedures, and supports needed during times of crisis that change the ways that people work. The similarities and differences among countries may be used to provide guidance for global recommendations specific to supporting working age residents.

## Conclusion

As suggested from this study, the benefits of working during this time period—even working remotely—includes lower emotional and overall loneliness, higher psychosocial well-being and quality of life, and less psychological distress overall. While participants who were not employed reported poorer mental health results related to emotional and overall loneliness, psychosocial well-being, quality of life, and psychological distress overall, it will be important to assess over time, if the economic aid implemented in each country to address employment loss will improve these outcomes.
